# The Novel *Enterococcus* Phage vB_EfaS_HEf13 Has Broad Lytic Activity Against Clinical Isolates of *Enterococcus faecalis*

**DOI:** 10.3389/fmicb.2019.02877

**Published:** 2019-12-17

**Authors:** Dongwook Lee, Jintaek Im, Hongjun Na, Sangryeol Ryu, Cheol-Heui Yun, Seung Hyun Han

**Affiliations:** ^1^Department of Oral Microbiology and Immunology, DRI, and BK21 Plus Program, School of Dentistry, Seoul National University, Seoul, South Korea; ^2^Department of Agricultural Biotechnology, Research Institute for Agriculture and Life Sciences, Center for Food and Bioconvergence, Seoul National University, Seoul, South Korea

**Keywords:** phage, *Enterococcus* phage HEf13, *Enterococcus faecalis*, phage infection protein of *Enterococcus faecalis*, whole genome analysis, human dentin

## Abstract

*Enterococcus faecalis* is a Gram-positive, facultative anaerobic bacterium frequently found in the gastrointestinal tract, oral cavity, and periodontal tissue. Although it is considered a commensal, it can cause bacteremia, endocarditis, endodontic infections, and urinary tract infections. Because antibiotics are cytotoxic not only to pathogens, but also to health-beneficial commensals, phage therapy has emerged as an alternative strategy to specifically control pathogenic bacteria with minimal damage to the normal flora. In this study, we isolated a novel phage, *Enterococcus* phage vB_EfaS_HEf13 (phage HEf13), with broad lytic activity against 12 strains of *E. faecalis* among the three laboratory strains and 14 clinical isolates of *E. faecalis* evaluated. Transmission electron microscopy showed that phage HEf13 has morphological characteristics of the family *Siphoviridae*. Phage HEf13 was stable at a wide range of temperature (4–60°C) and showed tolerance to acid or alkaline (pH 3–12) growth conditions. Phage HEf13 had a short latent period (25 min) with a large burst size (approximately 352 virions per infected cell). The lytic activity of phage HEf13 at various multiplicities of infection consistently inhibited the growth of diverse clinical isolates of *E. faecalis* without any lysogenic process. Moreover, phage HEf13 showed an effective lytic activity against *E. faecalis* on human dentin *ex vivo* infection model. Whole genome analysis demonstrated that the phage HEf13 genome contains 57,811 bp of double-stranded DNA with a GC content of 40.1% and 95 predicted open reading frames (ORFs). Annotated functional ORFs were mainly classified into four groups: DNA replication/packaging/regulation, phage structure, host cell lysis, and additional functions such as RNA transcription. Comparative genomic analysis demonstrated that phage HEf13 is a novel phage that belongs to the *Sap6virus* lineage. Furthermore, the results of multiple sequence alignment showed that polymorphism of phage infection protein of *E. faecalis* (PIP_EF_) contributes to determine the host specificity of phage HEf13 against various *E. faecalis* strains. Collectively, these results suggest that phage HEf13 has characteristics of a lytic phage, and is a potential therapeutic agent for treatment or prevention of *E. faecalis*-associated infectious diseases.

## Introduction

*Enterococcus* is a genus of Gram-positive, non-spore-forming bacteria that are frequently found in the oral cavity, gastrointestinal tract, and vagina (Jett et al., 1994). Although most *Enterococcus* species are considered to be commensal in humans and animals (Gu et al., 2018), *Enterococcus faecalis* can act as an opportunistic pathogen causing bacteremia, meningitis, endocarditis, or urinary tract- and wound-related infections (Arias et al., 2010; Weiner et al., 2016). In humans, *E. faecalis* has been predominantly detected in the root canals of patients with post-treatment apical periodontitis or refractory apical periodontitis, suggesting an etiological role in progression of these diseases (Souto and Colombo, 2008; Vidana et al., 2011).

Antibiotics are widely used to treat and prevent diseases caused by various pathogenic bacteria. However, the emergence of antibiotic resistant *E. faecalis*, such as vancomycin-resistant *E. faecalis* (VRE), has diminished the clinical usefulness of antibiotics (O’Driscoll and Crank, 2015). On the one hand, because most strains of *E. faecalis* isolated from root canal and periodontal infections show a high level of resistance to antibiotics including tetracycline and erythromycin used in dental procedures, antibiotic options for endodontic treatment are limited (Pinheiro and Mayer, 2014). On the other hand, indiscriminate use of antibiotics can result in disruption of normal gut microflora, and can negatively affect human health by promoting the evolution of antibiotic resistance in the microbiome and compromising immune homeostasis (Cho and Blaser, 2012; Francino, 2015).

There is currently renewed interest in phage therapy using lytic phages, also called viruses, as a potential alternative strategy to control pathogenic bacteria as this strategy overcomes the limitations of antibiotics (Golkar et al., 2014). Unlike antibiotics, phages are highly specific for their target pathogen and thus do not damage the normal flora (Moelling et al., 2018). Phages have many other advantages, including ease of isolation and better efficacy than antibiotics at destroying bacteria in biofilms (Khalifa et al., 2016). Moreover, many clinical trials have shown that the use of phages is safe without side-effects in both humans and animals (Burrowes et al., 2011).

Based on previously published studies, a total of 22 lytic phages targeting *E. faecalis* (14 *Siphoviridae*, 6 *Myoviridae*, and 2 *Podoviridae* phages) have been isolated from various sources, including sewage, animal farmyard effluents, and human feces (Bonilla et al., 2010; Bolocan et al., 2019; Lossouarn et al., 2019). Although previous studies have demonstrated the therapeutic potency of newly isolated phages for targeting *E. faecalis*, practical use of these phages has been limited by their relatively narrow host range against clinically isolated *E. faecalis* strains (Rigvava et al., 2013; Zhang et al., 2013; Ladero et al., 2016; Cheng et al., 2017; Rahmat Ullah et al., 2017; Wang et al., 2018). In addition, despite the importance of *E. faecalis* in the pathogenesis of recurrent and incurable periodontitis, curative efficiency of *E. faecalis* phages has not been evaluated against *E. faecalis* strains isolated from the oral cavity. Therefore, we focused on isolation, characterization, and genomic analysis of a novel *E. faecalis* lytic phage against clinically isolated *E. faecalis* strains from the oral cavity.

## Materials and Methods

### Bacterial Strains and Culture Conditions

A total of 17 strains of *E. faecalis*, including 14 clinical isolates and three laboratory strains, were used to determine the host range of phage HEf13. As shown in Table 1, three laboratory strains of *E. faecalis* and two laboratory strains of *E. faecium* were obtained from the Korean Agricultural Culture Collection (KACC, Wanju, Republic of Korea) and 14 clinically isolated *E. faecalis* strains, recovered from blood, dental plaques, mucormycosis, pus, urine (VRE), and the vagina were provided by the Korean Collection for Oral Microbiology (KCOM, Gwangju, Republic of Korea), Korean Collection for Type Cultures (KCTC, Jeongeup, Republic of Korea), or the National Culture Collection of Pathogens (NCCP, Chungju, Republic of Korea). All strains of *E. faecalis* were grown in brain heart infusion broth (BHI broth, Difco Laboratories Inc., Franklin Lakes, NJ, United States) at 37°C with shaking under aerobic conditions.

**TABLE 1:** Information of *E. faecalis* strains and the host specificity of phage HEf13.

***Enterococcus***	**Plaque formation^a^**	**Source^b^**
*Enterococcus faecalis*		
KACC 11304	T	KACC, type strain
KACC 11859	–	KACC, type strain
KACC 13807	–	KACC, type strain
KCOM 1083	C	KCOM, clinical isolate (dental plaque)
KCOM 1161	C	KCOM, clinical isolate (dental plaque)
KCOM 1162	C	KCOM, clinical isolate (dental plaque)
KCOM 2816	T	KCOM, clinical isolate (mucormycosis)
KCOM 2823	T	KCOM, clinical isolate (mucormycosis)
KCTC 5291	C	KCTC, clinical isolate (dental plaque)
KCTC 5292	C	KCTC, clinical isolate (dental plaque)
NCCP 14623	C	NCCP, clinical isolate (pus)
NCCP 14624	–	NCCP, clinical isolate (blood)
NCCP 14673	C	NCCP, clinical isolate (vagina)
NCCP 15611	–	NCCP, clinical isolate (blood)
NCCP 16130	T	NCCP, clinical isolate (VRE, urine)
NCCP 16131	–	NCCP, clinical isolate (VRE, urine)
NCCP 16132	T	NCCP, clinical isolate (VRE, urine)
*Enterococcus faecium*		
KACC 10782	–	KACC, type strain
KACC 11954	–	KACC, type strain

*^*a*^C, clear-plaques; T, turbid-plaques; –, no plaques and susceptibility. ^*b*^KACC, Korean Agricultural Culture Collection; KCOM, Korean Collection for Oral Microbiology; KCTC, Korean Collection for Type Cultures; NCCP, National Culture Collection of Pathogens.*

### Phage Isolation

Raw sewage water was collected from a local sewerage system (Seoul, Republic of Korea) and used as a source of *E. faecalis* phages. Phage isolation was performed as previously described (Lee et al., 2016). Briefly, sewage sample (10 mL) was homogenized with 40 mL sodium chloride–magnesium sulfate buffer [SM buffer; 100 mM NaCl, 10 mM MgSO_4_⋅7H_2_O, and 50 mM Tris–HCl (pH 7.5)] containing 1% chloroform. After centrifugation at 10,000 × *g* and 4°C for 10 min, supernatants were collected and then filtered through 0.22 μm pore-size membrane filters (Corning Inc., Corning, NY, United States). Supernatants (10 mL) were mixed with an equal volume of 2× concentrated BHI broth and incubated with 1% of overnight culture of 12 *E. faecalis* strains mixture with shaking at 37°C for 12 h. The suspension was harvested by centrifugation at 10,000 × *g* at 4°C for 10 min and filtered to remove bacterial debris. Then, supernatants diluted 10–1,000 times with BHI broth were spotted on molten 0.4% BHI soft agar containing 1 × 10^8^ CFU/mL of *E. faecalis*. The next day, clear phage plaques were picked from the BHI agar plates and the phages were resuspended in SM buffer. These phage plaque isolation and phage resuspension steps were repeated three times to obtain individual phages with high purity.

### Phage Propagation, Purification, and Titration

Propagation and purification of the phages were performed as described previously with minor modifications (Sambrook and Russell, 2001). For phage propagation, early log phase cultures of *E. faecalis* KCOM 1162 (OD_600_ = 0.35) were infected with phage and incubated with vigorous shaking at 37°C for 5 h. After centrifugation at 12,000 × *g* at 4°C for 10 min, supernatants containing phages were filtered using bottle-top vacuum filters with a 0.22-μm pore-size membrane (Merck Millipore, Burlington, MA, United States). Following the propagation step, filtrates were mixed with 1 M NaCl containing 10% polyethylene glycol 6000 (Junsei Chemical Co., Tokyo, Japan) and incubated overnight at 4°C. Precipitated phages were then harvested by centrifugation at 12,000 × *g* for 20 min at 4°C and resuspended in 2 mL SM buffer. Resuspended phages were subjected to density gradient ultracentrifugation (CP100NX, Hitachi, Tokyo, Japan) with different cesium chloride steps (density for each step = 1.3, 1.45, 1.5, and 1.7 g/mL) at 111,000 × *g* at 4°C for 2 h. Finally, purified phages were collected from the band corresponding to viral particles and dialyzed in standard dialysis buffer [10 mM NaCl, 10 mM MgCl_2_, 50 mM Tris–HCl (pH 8.0)] at 4°C for 2 h. To titrate the purified phages, serially diluted phages (10^–1^–10^–9^ folds) were dropped on an *E. faecalis* KCOM 1162 (1 × 10^9^ CFU/mL) lawn. Plaques were examined the next day and plaque-forming units (PFUs) were calculated based on the minimal dilution rate of phages generating plaques.

### Determination of Host Range

The host range of purified phages was determined by spotting assay (Kropinski et al., 2009) against the 17 strains of *E. faecalis* (Table 1). Briefly, 100 μL of each freshly cultured *E. faecalis* strain was mixed with 6 mL of warm 0.4% BHI soft agar and overlaid on previously prepared 1.5% BHI agar plates. After agar hardening, a 10 μL aliquot of purified phages at 1 × 10^9^ PFU/mL was spotted on each agar plate and the plates were incubated at 37°C overnight. The next day, bacterial sensitivity to phages was estimated by the clarity of plaques on each plate.

### Bacterial Challenge Assay

Among the 17 strains of *E. faecalis* tested in the host range assay, 12 strains sensitive to the purified phages based on the results of host range analysis were chosen for the bacterial challenge assay (Xie et al., 2018). One percent of an overnight culture of each *E. faecalis* strain was inoculated in triplicate on a 96-well plate and the plate was cultured at 37°C with shaking until the optical density at 600 nm (OD_600_) reached 0.2 (∼1 × 10^8^ CFU/mL). Bacteria were infected with the purified phages at various multiplicities of infection (MOI) ranging from 0.01 to 1, and incubated at 37°C with shaking for 0–12 h. Bacterial growth was assessed by measuring OD_600_ using a spectrophotometer every 2 h for up to 12 h. *E. faecalis* strains not infected with phages were used as controls for this assay. To determine whether phage HEf13 is a temperate or lytic phage, *E. faecalis* KCOM 2816 strain (1 × 10^8^ CFU/mL) at early mid log phase was infected with phage HEf13 (MOI 1) for 16 h. The infected bacteria were inoculated on 96-well plate by 1% (w/v) and incubated at 37°C for an additional 2 h. After the incubation, cultured bacteria were reinfected with phage HEf13 (MOI 0.1 and 1) in the presence or absence of mitomycin C (MMC) at 5 μg/mL (Sigma–Aldrich, St. Louis, MO, United States) (Stevens et al., 2009). Bacterial growth was measured every 2 h for up to 12 h using a spectrophotometer.

### Preparation of Human Dentin Slices

Experiments using human dentin slices were approved by the Institutional Review Board of Seoul National University Dental Hospital, Seoul, Republic of Korea (CRI 17010). Extracted human single-rooted pre-molars were sonicated in 0.5% sodium azide with an ultrasonic scaler (SH-2140; Saehan-Sonic, Seoul, Republic of Korea). The roots were sliced into a size of 500 μm with an Isomet precision saw (Buehler, Lake Bluff, IL, United States). The slices were sequentially treated with 17% EDTA (Sigma–Aldrich) for 5 min, 2.5% sodium hypochlorite (Sigma–Aldrich) for 5 min, and 5% sodium thiosulfate (Sigma–Aldrich) for 5 min, followed by autoclave-sterilization for 15 min at 121°C.

### *Ex vivo* Analysis of Phage Lytic Activity

The lytic activity of phage HEf13 against *E. faecalis* on human dentin slice was examined by scanning electron microscope (SEM) analysis as previously described (Kim et al., 2019). Briefly, *E. faecalis* (1 × 10^8^CFU/mL) was grown on dentin slices in the presence or absence of phage HEf13 (MOI 0.1) in BHI medium at 37°C for 12 h. Attached bacteria on dentin slice were rinsed with phosphate-buffered saline (PBS) and prefixed with a 2.5% glutaraldehyde and 2% paraformaldehyde (pH 7) at 4°C overnight. After washing with PBS, the dentin slices were fixed with 1% osmium tetroxide for 90 min, then washed with distilled water, and subsequently dehydrated by gradual increase of ethanol concentrations (70–100% for 15 min). After drying with hexamethyldisilazane and coating with gold sputter, samples were visualized by SEM (S-4700, Hitachi, Tokyo, Japan).

### Morphological Analysis by Electron Microscopy

Morphology of the purified phages was examined by transmission electron microscopy (TEM) analysis. The glow-discharge step to prepare Formvar/carbon-coated copper grids was performed at 15 mA and 0.26 mBar for 30 s using a glow discharge cleaning system (PELCO easiGlow; Ted Pella Inc., Redding, CA, United States). Purified phage stock (approximately 3 × 10^7^ PFU) was dropped onto the prepared Formvar/carbon-coated copper grids and incubated at room temperature for 1 min. *E. faecalis* KCOM 1162 (1 × 10^8^ CFU/mL) incubated with phages (1 × 10^10^ PFU/mL) at room temperature for 10 min was collected by centrifugation and resuspended in SM buffer. Bacteria were also dropped onto the prepared Formvar/carbon-coated copper grids and after a 1 min incubation, the grids were negatively stained with 2% uranyl acetate and electron micrographs of phages were obtained by TEM (Libra 120 model; Zeiss, Oberkochen, Germany operated at an accelerating voltage of 80 kV). Identification and classification of phages was conducted according to the guidelines of the International Committee on the Taxonomy of Viruses (ICTVs).

### One-Step Growth Curve Analysis

One-step growth experiments were conducted as described previously with a slight modification (Parasion et al., 2012). When the culture of *E. faecalis* KCOM 1162 reached early log phase (OD_600_ = 0.35), bacteria were harvested by centrifugation at 10,000 × *g* at 4°C for 10 min and resuspended in fresh BHI broth. Bacteria were infected with purified phages at a MOI of 0.01 and incubated at room temperature for 5 min. After centrifugation and removal of the supernatant containing unbound phages, pellets were resuspended in 50 mL fresh BHI medium and incubated at 37°C with shaking for up to 90 min. Samples were taken every 5 min, serially diluted, and plated on *E. faecalis* KCOM 1162 lawn for phage titration. Burst sizes of phages were calculated as the ratio of the average phage titer value at plateau phase to that at latent phase (Svab et al., 2018).

### Thermal and pH Stability

Stability of the phages to temperature and pH was assessed by phage titration after incubation in SM buffer at different temperature or pH values (Park et al., 2017). Briefly, 1.5 mL tubes containing the same amount of the purified phages (1 × 10^8^ PFU/mL) were incubated at various temperatures ranging from 4 to 70°C, or under different pH conditions (SM buffer at pH 2–12 adjusted by NaOH or HCl) at 37°C for 12 h. After incubation, phage titers were determined using *E. faecalis* KCOM 1162 as described above.

### Whole Genome Sequencing and Bioinformatic Analysis

Genomic DNA from purified phages was extracted using the Norgen phage DNA isolation kit (Norgen Biotek Corp., ON, Canada) according to the manufacturer’s instruction. Sequencing libraries with single index were prepared using the TruSeq Nano DNA library prep kit (Illumina, San Diego, CA, United States) and then sequenced on the Illumina MiSeq sequencing platform (Illumina, San Diego, CA, United States) with paired-end 300 nucleotide reads. Raw reads were trimmed and *de novo* assembled using the CLC Genomic Workbench program (Version 10.0.1; QIAGEN Inc., Hilden, Germany). Annotation of the specific function of ORFs was conducted using rapid annotations of subsystems technology (RAST) and the BLASTP database (Altschul et al., 1997; Aziz et al., 2008). Artemis (version 16; Sanger Institute, Cambridge, United Kingdom) was used to visualize and browse the genome and annotate ORFs (Carver et al., 2008). The presence of tRNA-encoding genes was determined using the tRNAscan-SE database (Lowe and Chan, 2016). A circular representation of the genome of phage HEf13 was visualized using the CGView server database (Stothard and Wishart, 2005). Phage virulence factor analysis was conducted using the Virulence Searcher database (Underwood et al., 2005). Comparative analysis of genome sequences and visualization of the genomes of phage HEf13 and other *Enterococcus* phages was performed using BLASTN and BLASTP databases and the EasyFig program (Version 2.2; Beatson Microbial Genomic Lab, Brisbane, Australia) (Sullivan et al., 2011).

### Comparative Phylogenetic Analysis

Full length amino acid (aa) sequences of the phage portal protein (511 aa), tail fiber protein (1,330 aa), and DNA methyltransferase (150 aa) of phage HEf13 were aligned with those of nine *Enterococcus* phages in the *Sap6virus* genus and *Listeria* phages showing high similarity using the ClustalW Multiple sequence alignment (MSA) module in the BioEdit Sequence Alignment Editor program (Ibis Therapeutics, Carlsbad, CA, United States) (Larkin et al., 2007). After alignment, phylogenetic trees for each of the three phage proteins were generated by the Neighbor-joining method using Molecular Evolutionary Genetics Analysis 7 (MEGA7) software (Pennsylvania State University, State College, PA, United States) (Tamura et al., 2013) and groupings were estimated by bootstrap analysis (1,000 replication).

### Analysis of Phage Infection Protein of *E. faecalis* (PIP_EF_) Polymorphism

Polymorphism of the variable region of PIP_EF_ for *E. faecalis* strains used in the current study was analyzed to examine correlation between the host specificity of phage HEf13 and PIP_EF_ as previously described (Duerkop et al., 2016). Briefly, genomic DNA was extracted from 17 strains of *E. faecalis* using a commercially available kit according to the manufacturer’s instruction (iNtRON Biotechnology, Seongnam, Republic of Korea). The genomic DNAs were amplified by PCR under the following conditions: denaturation at 95°C for 30 s and amplification via 28 cycles of 50°C for 3 min and 72°C for 3 min. PCR products were purified using a PCR/Gel purification kit (Bioneer, Daejeon, Republic of Korea) and sequenced at Cosmo Genetech (Seoul, Republic of Korea). MSAs of homologs were analyzed using Geneious 6.0.5. software (Biomatters Ltd., Auckland, New Zealand). Phylogenetic tree of PIP_EF_ homologs was constructed by Neighbor-Joining method (1,000 bootstrap) (Tamura et al., 2013).

## Results

### Isolation, Host Range Determination, and Lytic Activity of Phage HEf13

**FIGURE 1:**
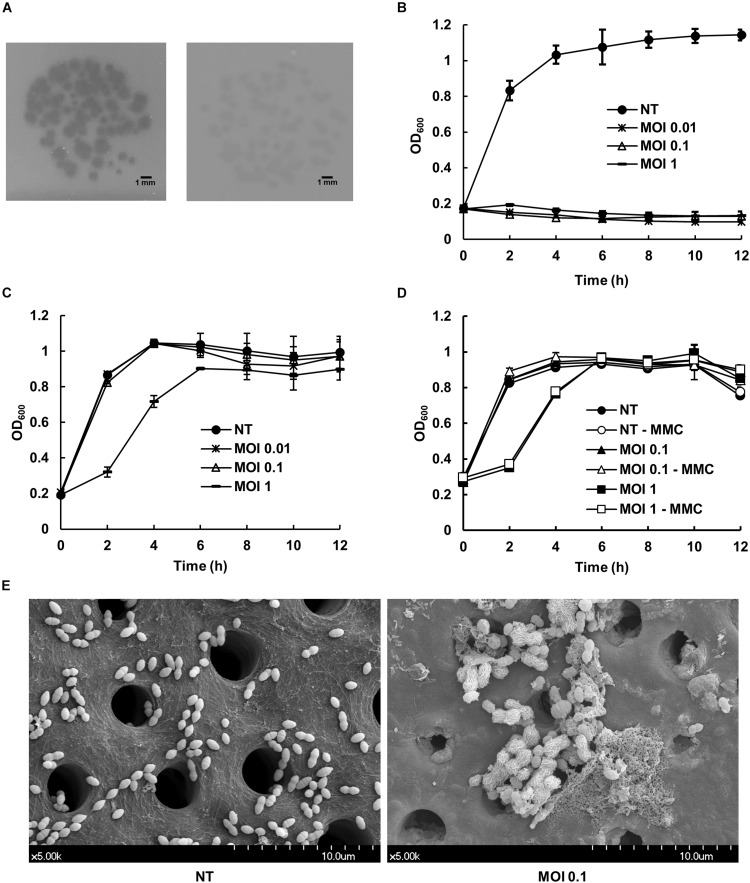
Effect of newly isolated *Enterococcus* phage vB_EfaS_HEf13 (phage HEf13) on the growth of *E. faecalis*. **(A)** Phage HEf13 suspension (1 × 10^4^ PFU/mL) was spotted on *E. faecalis* KCOM 1162 (clear-plaque, left) or KACC 11304 (turbid-plaque, right) lawn and incubated at 37°C overnight. Plaque formation by phage HEf13 was photographed. Scale bar indicates 1 mm. **(B,C)** Representative *E. faecalis* strains showing clear- (**B**; KCOM 1162) or turbid-plaque formation (**C**; KCOM 2816) by phage HEf13 were chosen for the bacterial challenge assay. Each *E. faecalis* strain was infected with phage HEf13 at various MOI (0.01–1) and incubated at 37°C for 0–12 h. **(D)** Turbid-plaque-forming *E. faecalis* strain (KCOM 2816) was infected with phage HEf13 (MOI 1) for 16 h and the infected bacteria were then grown to early mid-log phase. After the culture, the infected bacteria were cultured in the presence or absence of phage HEf13 (MOI 0.1 or 1) and MMC at 5 μg/mL. Bacterial growth at each indicated time point was measured by spectrophotometer at 600 nm. Values are means ± standard deviations from triplicates of each treatment. **(E)** Dental isolate of *E. faecalis* strain (KCOM 1162) was inoculated on human dentin slices in the presence or absence of phage HEf13 (MOI 0.1) for 12 h. Then, the dentin slices were fixed and images were obtained by SEM (magnification: 5,000×). Scale bar indicates 10 μm with 1 μm ticks. MMC, mitomycin C; MOI, multiplicity of infection; NT, non-treatment.

*E. faecalis* phage vB_EfaS_HEf13 (phage HEf13) was newly isolated from sewage water of a local sewerage system in Seoul, Republic of Korea. The host range of phage HEf13 was assessed using a spotting assay against a total 17 strains of *E. faecalis* (three laboratory strains and 14 clinically isolated strains including three VRE strains), and two laboratory strains of *E. faecium*. Phage HEf13 formed clear- or turbid-plaques in 12 *E. faecalis* strains, including all dental isolates of *E. faecalis* (Figure 1A and Table 1). However, phage HEf13 cannot recognize and lyse *E. faecium*, suggesting that phage HEf13 is specific only to *E. faecalis*, but not to other *Enterococcus* species. To examine the lytic activity of phage HEf13, *E. faecalis* strains where clear- or turbid-plaques were observed in the spotting assay were subjected to bacterial challenge assays at various MOI for up to 12 h. Similar to the pattern of host range determination, phage HEf13 showed strong lytic activity against *E. faecalis* where clear-plaque formation was observed in the previous spotting assay, but not the strains where turbid-plaque formation was observed (Figures 1B,C and Supplementary Figures S1A–J). However, the results showed a possibility that turbid-plaque formation by phage HEf13 may result from its lysogenic property. Therefore, in order to determine whether the phage is lytic or lysogenic, we examined the growth of *E. faecalis* infected with phage HEf13 in the presence of MMC, which is a lysogenic phage inducer (Stevens et al., 2009). As shown in Figure 1D. *E. faecalis* infected with phage HEf13 was not affected by MMC treatment, implying that the turbid-plaque formation by phage HEf13 was not due to a lysogenic process of phage HEf13. Furthermore, we evaluated the effect of phage HEf13 against *E. faecalis* using an *ex vivo* human dentin infection model. When *E. faecalis* on dentin slice infected with phage HEf13 was visualized using a SEM, *E. faecalis* was completely eradicated (Figure 1E). These results suggest that phage HEf13 is a potential therapeutic agent to treat diseases associated with *E. faecalis* infection such as refractory apical periodontitis.

### Morphology of Phage HEf13

**FIGURE 2:**
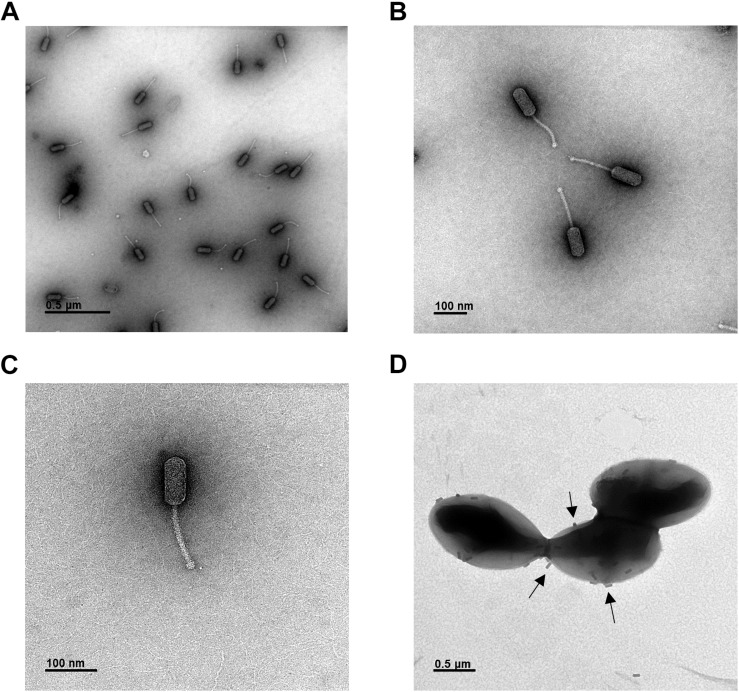
Phage HEf13 belongs to the *Siphoviridae* family. **(A–C)** Phage HEf13 (1 × 10^8^ PFU) was dropped onto a Formvar/carbon-coated copper grid and then negatively stained with 2% uranyl acetate. **(D)** *E. faecalis* KCOM 1162 (1 × 10^8^ CFU/mL) incubated with phage HEf13 (1 × 10^10^ PFU/mL) was dropped onto copper grids and stained with 2% uranyl acetate. Electron micrographs of phage HEf13 were obtained by TEM at **(A)** 35,000×, **(B,C)** 100,000×, and **(D)** 35,000× magnification. Identification and classification of phages was conducted according to ICTV guidelines. Scale bar indicates 100 nm **(B,C)** or 0.5 μm **(A,D)**. Arrows in panel **(D)** show phage HEf13 binding to the cell wall membrane of *E. faecalis* KCOM 1162.

Morphological analysis of phage HEf13 and its adsorption to *E. faecalis* was examined by TEM. Phage HEf13 has a prolate head with a long non-contractile tail (Figures 2A–C). Head diameter is 38.4 ± 2.4 nm with a length of 95.6 ± 1.7 nm, and tail length is 135.2 ± 4.7 nm (Figures 2A–C). TEM images of *E. faecalis* KCOM 1162 infected with phage HEf13 demonstrated adsorption of phages to the surface of *E. faecalis* as marked by the arrows in Figure 2D. Taken together, these results indicate that phage HEf13 belongs to the *Siphoviridae* family in the order *Caudovirales* according to the ICTV guidelines (Fauquet et al., 2005).

### Latent Period and Burst Size of Phage HEf13

**FIGURE 3:**
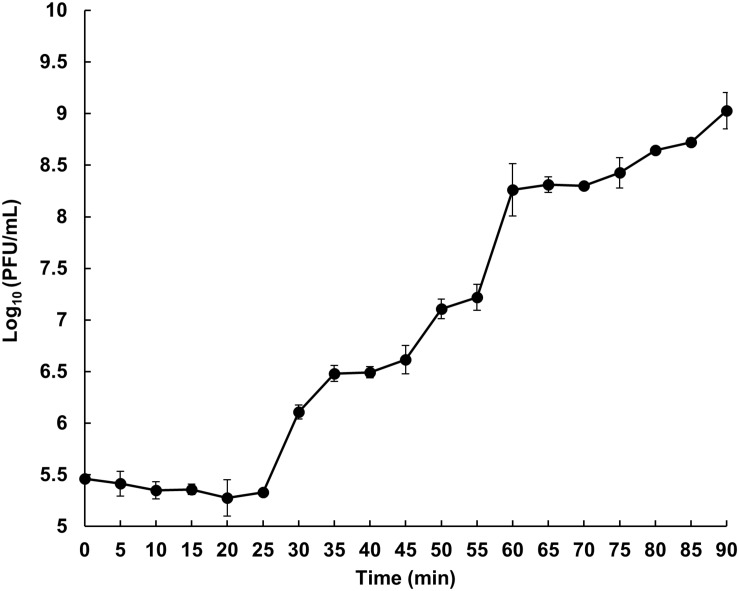
Phage HEf13 has a relatively short latent period and enhanced large burst size against *E. faecalis*. *E. faecalis* KCOM 1162 was infected with Phage HEf13 at an MOI of 0.01 and incubated for 5 min. After removing unbound phages by centrifugation, bacteria were incubated at 37°C up to 90 min. Samples were taken every 5 min and subjected to phage titration by spotting assay as described in the section “Materials and Methods.” Values are means ± standard deviations from triplicates of each time point.

The latent period and burst size of phage HEf13 were measured by one-step growth curve analysis against *E. faecalis* KCOM 1162. The latent period of phage HEf13, calculated as the time interval between adsorption and the beginning of the first burst, was approximately 25 min (Figure 3). The rise period of phage HEf13 was about 30 min and mean burst size, defined as the mean phage titer value at plateau phase divided by that of the latent phase, was approximately 352 virions per infected cell. These results indicate that phage HEf13 has a short latent period and long rise period with a large burst size, accounting for its strong lytic activity against *E. faecalis*.

### Stability of Phage HEf13 to Different Temperature and pH Conditions

**FIGURE 4:**
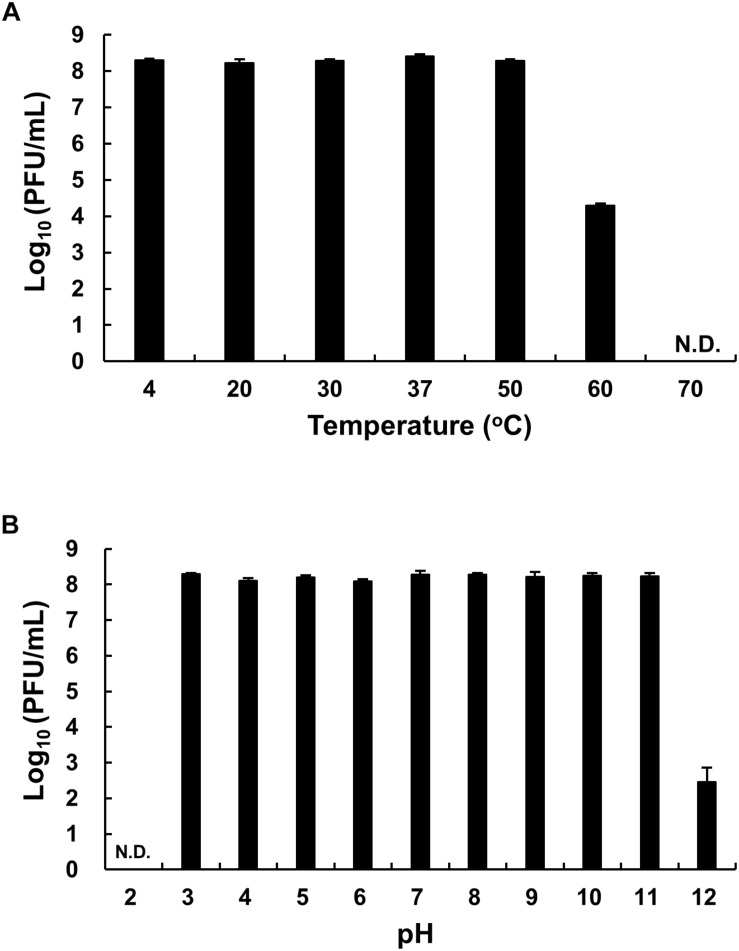
Phage HEf13 is stable over a broad range of temperature and pH values. **(A)** Phage HEf13 (1 × 10^8^ PFU/mL) was incubated at various temperatures ranging from 4 to 70°C for 12 h. **(B)** Phage HEf13 (1 × 10^8^ PFU/mL) was also incubated at different pH values ranging from 2 to 12 at 37°C for 12 h. Then, phage titers were determined by spotting assay as described in the section “Materials and Methods.” Values are presented as log values of the ratio of phage titers in each treatment to that of the initial phage titer. Values are means ± standard deviations from triplicates of each treatment. N.D., not detected.

The thermal stability of phage HEf13 was evaluated by phage titration assay after phage incubation at various temperatures ranging from 4 to 70°C for 12 h. While no loss was observed in phage titers at temperatures ranging from 4 to 50°C, a phage titer of <60% was observed at 60°C. Furthermore, no phage titer was detected when phage HEf13 was incubated at 70°C (Figure 4A). Phage HEf13 tolerated a broad range of pH values from 3 to 12. However, no phage activity was noted under extremely acidic conditions (pH 2) (Figure 4B). These data show that phage HEf13 can tolerate a broad range of temperature and pH values.

### Genomic Characterization of Phage HEf13

**FIGURE 5:**
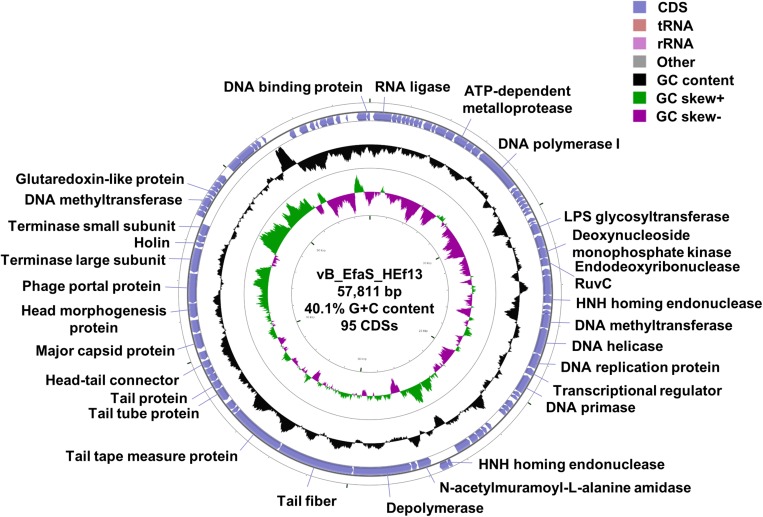
Genome map of phage HEf13 and its genetic characteristics. The whole genome sequence of phage HEf13 was determined using the Illumina Miseq platform and circular genome visualization of phage HEf13 was performed using the CGView server database. Annotation of the specific function of ORFs was conducted using RAST and the BLASTP database, and the complete genome sequence and annotation of ORFs were handled using the Artemis program. The second inner circle with the green and purple histogram shows the GC skew +/–, while the fourth inner circle with the black histogram indicates the GC content. The outer circle indicates the predicted ORFs of phage HEf13 together with their putative functions.

Whole genome sequence analysis showed that the genome of phage HEf13 (GenBank Accession Number: MH618488) is 57,811 bp in length with 95 predicted ORFs and one tRNA gene, a GC content of 40.03%, and the following nucleotide composition: G (10,737 bp, 18.57%), C (12,405 bp, 21.45%), A (15,765 bp, 27.26%), and T (18,904 bp, 32.69%) (Figure 5). Average gene length was 532 bp, with a range of 116–3,992 nucleotides, and gene coding percentage was 87.4%. Among the 95 putative ORFs, 15 ORFs were on the positive strand while the other 80 ORFs were on the negative strand. Only 30 ORFs (31.5%) were predicted to be functional proteins, whereas 65 ORFs (68.4%) were annotated as hypothetical proteins (Supplementary Table S1). Annotation of the function of the 30 ORFs predicted as functional proteins by RAST and BLASTP analyses revealed four major functional groups: DNA replication/packaging/regulation, phage structure, host cell lysis, and additional function. The DNA replication/packaging/regulation module comprised 16 genes, suggesting that phage HEf13 has a host-independent DNA replication/packaging/regulation system with the following components: DNA replication proteins (DNA polymerase I, DNA primase, DNA replication protein, and replicative DNA helicase), packaging proteins (glutaredoxin-like protein, phage portal protein, phage terminase large subunit, and phage terminase small subunit), and regulation proteins (crossover junction endodeoxyribonuclease RuvC, cytidine deaminase, deoxynucleoside monophosphate kinase, DNA binding protein, DNA methyltransferase, HNH endonuclease, and HNH homing endonuclease). The phage structural module comprised seven genes required for host recognition and phage structural assembly: tail-associated proteins (phage tail protein, phage tail tube protein, tail fiber, and tail tape measure protein) and head-associated proteins (head morphogenesis protein, head-tail connector family protein, and major capsid protein). The host cell lysis gene module encoded cell-wall binding and cell lysis proteins (depolymerase, endolysin, and holin). In addition, four functional proteins had annotations of RNA transcription (RNA ligase and transcriptional regulator) or unidentified function (ATP-dependent metalloprotease and LPS glycosyltransferase). No lysogenic genes such as lytic repressor proteins, recombinases, excisionases, or integrases were identified, suggesting that phage HEf13 might be an obligate lytic phage only with a lytic cycle. Furthermore, virulence factor analysis of the phage HEf13 genome using the Virulence Searcher database revealed that none of the ORFs encode any functional proteins that can act as human virulence factors. Thus, our results suggest that phage HEf13 does not contain any possible pathogenic factors, and is therefore safe to use to treat *E. faecalis*-associated diseases.

### Comparative Genome Analysis of Phage HEf13 and Other *Enterococcus* Phages

**FIGURE 6:**
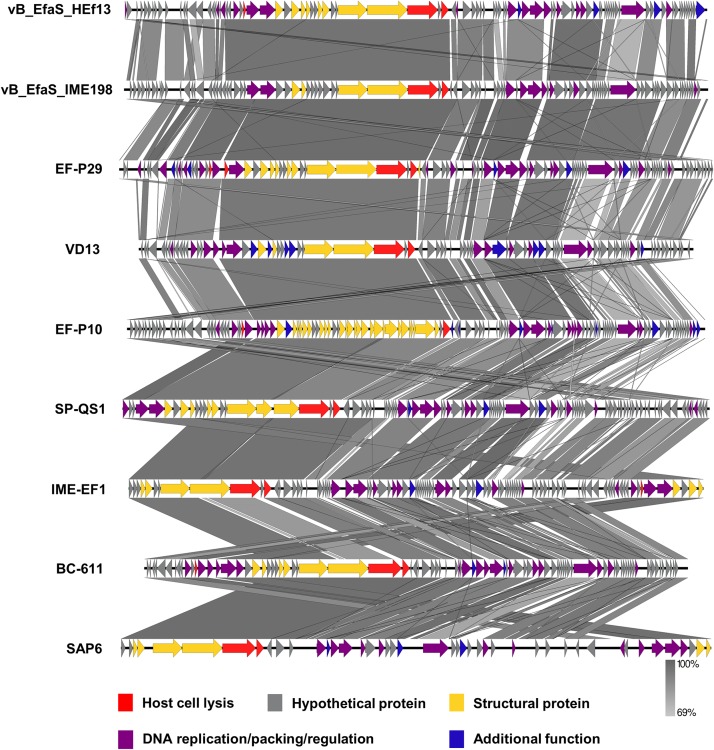
Phage HEf13 has high homology with phages in the *Sap6virus* genus of *Enterococcus* phages. Comparative genome analysis of phage HEf13 and eight other *Enterococcus* phages was performed using the EasyFig program. ORFs of phages were color-coded according to their predicted functions: host cell lysis (red), hypothetical protein (gray), structural protein (yellow), DNA replication/packing/regulation (purple), and additional function (blue). Genetic similarity profiles between phage HEf13 and other phages are presented in grayscale (percent homology).

**FIGURE 7:**
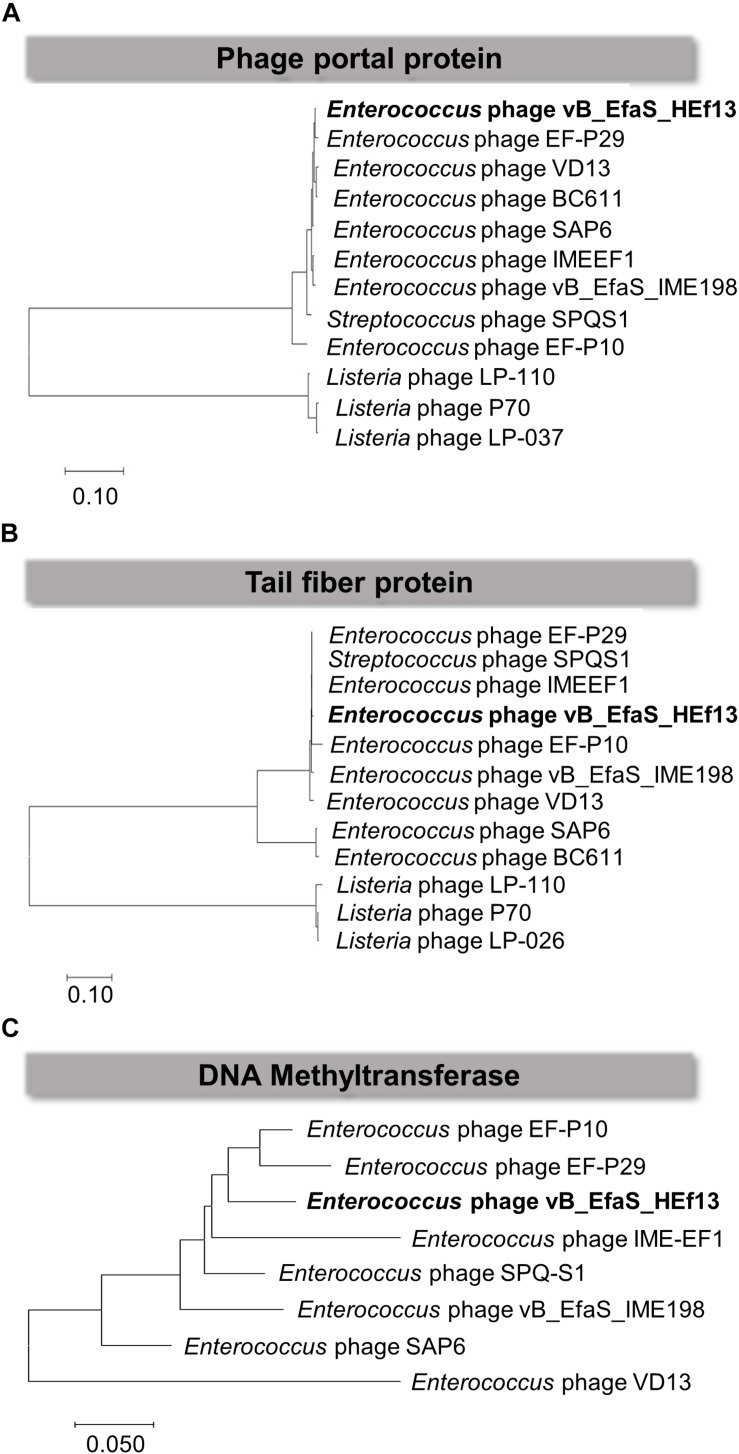
Phage HEf13 was classified as the *Sap6virus* lineage. The amino acid sequences of the phage portal protein, tail fiber protein, and DNA methyltransferase of phage HEf13 were compared with those of other phages using the BioEdit Sequence Alignment Editor program. A phylogenetic tree for each phage protein was generated using MEGA7 software. Scale bars represent a 10 **(A,B)** or 5% **(C)** difference in sequence similarity between phages.

Comparison of the nucleotide sequence of phage HEf13 with that of other *E. faecalis* phages using the BLASTN database revealed an average similarity of 95% between vB_EfaS_IME198 and phage EF-P29 (84–85% sequence coverage) belonging to the *Sap6virus* genus (Supplementary Table S1). To further analyze similarities and differences between phage HEf13 and other *Sap6virus* phages, we visualized comparative genome maps to assess similarities and differences in ORF organization. Most of the genes from phage HEf13 and other relative *Sap6virus* phages had a similar gene arrangement and were highly homologous. However, some genetic differences were observed in genes in the DNA replication/packing/regulation module (indicated in purple in Figure 6). Based on comparison of amino acid sequences using BLASTP, 77 of 95 ORFs in phage HEf13 shared 90–100% identity with ORFs of the other compared phages, but 18 ORFs, including the four unidentified ORFs, shared 0–89% identity (Supplementary Table S2). Interestingly, the four unidentified ORFs had no significant homology with any other sequences in *E. faecalis* phages. To determine the taxonomic affinity of phage HEf13, phylogenetic analysis using previously published genome sequences of eight other phages belonging to the *Sap6virus* genus was conducted. Phylogenetic trees constructed based on the amino acid sequences of phage portal protein, tail fiber protein, and DNA methyltransferase demonstrated that phage HEf13 clustered within the *Sap6virus* genus together with phages EF-P29, VD13, BC-611, SAP6, IME-EF1, vB_EfaS_IME198, SPQ-S1, and EF-P10 (Figures 7A–C). Collectively, the results of the phylogenetic analysis together with results of morphological – and comparative genome – analyses indicate that phage HEf13 belongs to the genus *Sap6virus* in the family *Siphoviridae*, order *Caudovirales*.

### Analysis of Polymorphism in the Potential Phage Receptor PIP_EF_

**FIGURE 8:**
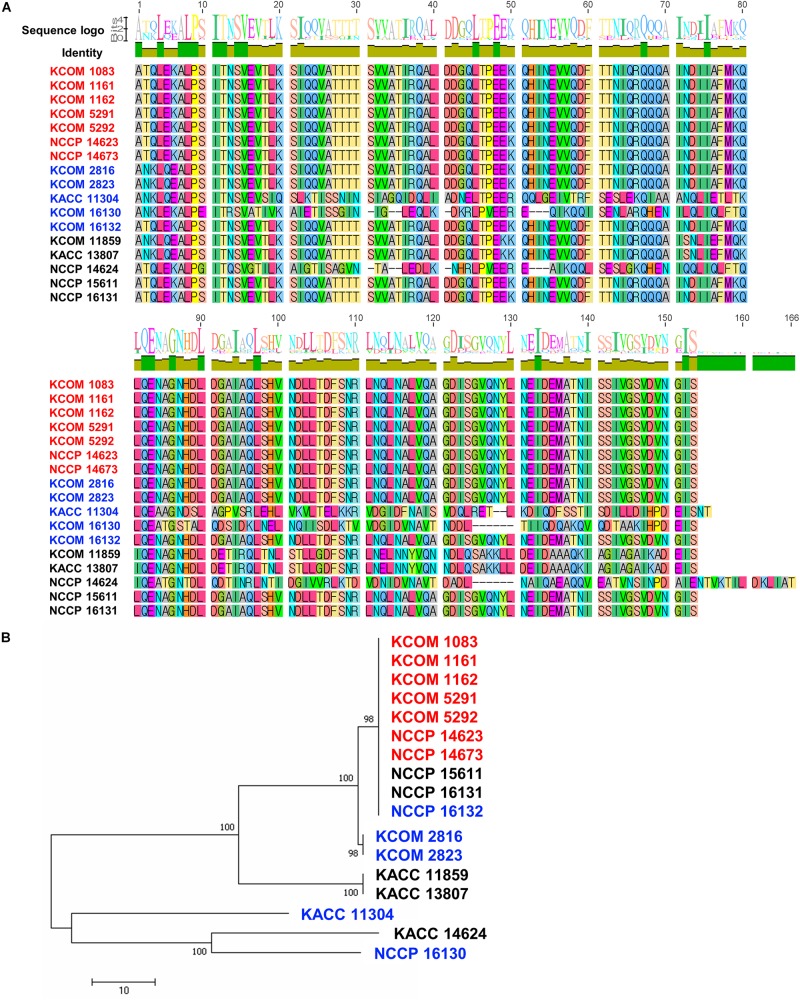
Polymorphism in the variable region of PIP_EF_ in *E. faecalis* strains is related with phage HEf13 host specificity. **(A)** Multiple amino acid sequence alignment of the PIP_EF_ variable region in 17 strains of *E. faecalis* was generated by Geneious 6.0.5 software. Difference in amino acid sequence of *E. faecalis* strains is presented in different colors. **(B)** Phylogenetic tree of PIP_EF_ homologs was constructed by Neighbor-joining method with 1,000 replicates. Scale bar represents a 10% difference in sequence similarity between homologs. Strains are indicated by color as follows: clear-plaque-forming strain (red), turbid-plaque-forming strains (blue), and phage-resistant strains (black).

To understand the host specificity of phage HEf13, we investigated host cell receptors for phage HEf13. Among various bacterial cell wall membrane proteins, the PIP_EF_ has recently been identified as a specific host cell receptor of *E. faecalis Siphoviridae* phages. Moreover, it has been reported that polymorphism in the variable region (covering amino acids 342–494) of PIP_EF_ affects the binding affinity of phages to the host bacteria, subsequently determining the host range of the phage (Duerkop et al., 2016). Therefore, we examined polymorphism in the variable region of PIP_EF_ by MSA for all *E. faecalis* strains used in the current study. As shown in Figure 8A, all of clear-plaque-forming strains have the same amino acid sequence in the variable region of PIP_EF_. However, most of turbid-plaque-forming strains and phage-resistant strains (7 of 10 strains), except for one turbid-plaque-forming strain and two phage-resistant strains, showed different sequences with those of clear-plaque-forming strains. Furthermore, phylogenetic tree of PIP_EF_ based on the results of MSA showed that cluster of clear-plaque-forming strains were clearly distinguished from that of turbid-plaque-forming strains and phage-resistant strains, except for three strains (NCCP 15611, 16131, and 16132) (Figure 8B). These results demonstrated that polymorphism of PIP_EF_ might be closely related with the host specificity of phage HEf13 to *E. faecalis*.

## Discussion

Antibiotic-resistant *E. faecalis* strains have decreased the utility of antibiotics in clinical settings, making the phage therapy a highly attractive alternative therapeutic strategy. In this study, we isolated a novel lytic *E. faecalis* phage belonging to the genus *Sap6virus* in the family *Siphoviridae*. Our results demonstrated that phage HEf13 has a relatively broader host spectrum than previously isolated *E. faecalis* lytic phages. Notably, the host range of phage HEf13 was 70.5% against all tested *E. faecalis* strains (12 out of 17 tested strains), which is broader than other *E. faecalis* lytic phages with the host range from 7.6 to 42.5% (Rigvava et al., 2013; Zhang et al., 2013; Ladero et al., 2016; Cheng et al., 2017; Rahmat Ullah et al., 2017; Wang et al., 2018).

Regarding burst size, whereas the average burst size of *E. faecalis* phages reported previously was typically 36–122 PFU/infected bacteria, phage HEf13 has an approximately threefold higher burst size of 352 PFU/infected bacteria (Rigvava et al., 2013; Cheng et al., 2017; Wang et al., 2018). Moreover, phage HEf13 has an average latent time of 25 min after infection, which is shorter than those of other *E. faecalis* phages (30–50 min) (Uchiyama et al., 2008; Cheng et al., 2017; Wang et al., 2018). In addition, phage HEf13 showed strong lytic activities against all tested dental clinical isolates of *E. faecalis* at a broad range of MOI from 0.01 to 1 (Figure 1B and Supplementary Figures S1A,B,E,F). Earlier *E. faecalis* phage studies tested the lytic activity of phages against laboratory strains or clinically isolated antibiotic resistant strains recovered from various clinical specimens, but not against clinical isolates from oral specimens (Parasion et al., 2012; Rigvava et al., 2013; Zhang et al., 2013; Cheng et al., 2017; Wang et al., 2018). Thus, to the best of our knowledge, this is the first study to demonstrate the potential utility of *E. faecalis* phages as dental therapeutic reagents for post-treatment apical or refractory apical periodontitis, which are conditions closely related to *E. faecalis* infection.

Phage HEf13 appeared to be highly stable to a broad range of temperatures (4–60°C) and pH values (3–12). While phage HEf13 maintained over 50% of its titer for a long-term incubation period (after 12 h) at 60°C (Figure 4A), other previously isolated *E. faecalis* phages maintained about 40% of their titer for a short-term incubation period (even after 1 h) at 45–60°C (Rigvava et al., 2013; Rahmat Ullah et al., 2017). Since little is known about the pH stability of *E. faecalis* phages, we compared the pH stability of phage HEf13 with those of other Gram-positive bacteria phages. Phage HEf13 titers were not affected by incubation at pH 11 and remained at around 30% at pH 12 (Figure 4B), whereas reported titers of other phages were almost completely abolished at pH 11 (Li and Zhang, 2014; Chen et al., 2016; Phothichaisri et al., 2018). These comparisons demonstrated that phage HEf13 is relatively more stable to high temperatures and strongly basic conditions than other phages. Furthermore, these findings suggest that phage HEf13 may be highly potent in clinical settings considering that *E. faecalis* can survive and grow under harsh environments such as a pH of 11 and temperature of 45°C (McHugh et al., 2004; Foulquie Moreno et al., 2006). These characteristics of phage HEf13 make it potentially ideal to use in combinatory treatment with alkaline disinfectants such as calcium hydroxide and sodium hypochlorite, which are commonly used to treat endodontic infection.

Comparative genome analysis revealed that the genome sequence of phage HEf13 is highly conserved with those of other tested *Sap6virus* phages. Among 77 ORFs that showed high similarity to the ORFs of other phages in this genus, we found two ORFs of phage HEf13 that may generate different host specificity and phage protection based on differences in amino acid sequence. While the receptor binding protein (RBP) in the phage tail apparatus of phage HEf13 (ORF55) has a negatively charged amino acid (443D), the other eight *Sap6virus* phages evaluated have an uncharged amino acid at same position (443N). Because RBP plays an important role in phage adsorption to host bacteria, this may result in a different host specificity of phage HEf13 to other phages in same genus. In fact, a recent study using point-mutated phages demonstrated that point mutation of an amino acid in the RBP (K653N) generated significant differences in host specificity (Le et al., 2013). Additionally, the sequence of DNA methyltransferase (ORF75), which protects phages from bacterial restriction modification system, differed by 8–16% between phage HEf13 and the other *Sap6virus* phages evaluated, suggesting differences in phage protection.

The host specificity of HEf13 against *E. faecalis* appears to be associated with the PIP_EF_ in light of the fact that all of the clear-plaque-forming strains possess the same amino acids sequence in the variable region of PIP_EF_ even though turbid-plaque-forming strains and phage-resistant strains were not differentially clustered in the phylogenetic tree. Remarkably, three strains belonging to turbid-plaque-forming strain (NCCP 16132) or phage-resistant strains (NCCP 15611 and 16131) were also clustered in the same clade with clear-plaque-forming strains (Figure 8B). Although further study is needed, it can be explained by two potential mechanisms based on the previous studies. First, Gram-positive bacterial cell-wall components, such as capsular polysaccharides, wall-teichoic acids, and lipoteichoic acids, could hide the phage receptor by masking them, subsequently making phages inaccessible to its receptor (Munsch-Alatossava and Alatossava, 2013; Ozaki et al., 2017). Second, the aforementioned bacterial cell wall components could also compete with the receptors for phage adsorption, thereby interfering with a successful phage infection (Seed, 2015).

So far, a total of 22 *E. faecalis* lytic phages have been isolated and their stability and efficacy against various clinical and non-clinical strains have been evaluated. The phages identified previously, however, have a relatively narrow host spectrum and low stability to broad temperature and pH ranges, limiting their practical usefulness. Here, we characterized a newly isolated phage, HEf13, and demonstrated that this phage is highly potent against various *E. faecalis* strains and has high lytic ability and stability. Furthermore, the high lytic activity of phage HEf13 against dental clinical isolates suggests that phage HEf13 may be effective as a dental therapeutic agent to treat recurrent or refractory apical periodontitis related to *E. faecalis* infection.

## Data Availability Statement

The raw datasets generated in this study can be found in GenBank, Accession Number: MH618488.

## Author Contributions

DL and SH designed the research. DL and HN carried out the experiments. DL, JI, and SH analyzed and interpreted the data. DL, JI, SR, C-HY, and SH prepared and reviewed the manuscript. All authors have read and accepted the final manuscript.

## Conflict of Interest

The authors declare that the research was conducted in the absence of any commercial or financial relationships that could be construed as a potential conflict of interest.
