# Supplementary material for: The Novel Enterococcus Phage vB_EfaS_HEf13 Has Broad Lytic Activity Against Clinical Isolates of Enterococcus faecalis
Source: Front Microbiol. 2019 Dec 17;10:2877. doi: 10.3389/fmicb.2019.02877 (PMC6927925; doi:10.3389/fmicb.2019.02877)
Supplement: Supplementary file 1 [file Presentation_1.pdf]

Supplementary figure 1

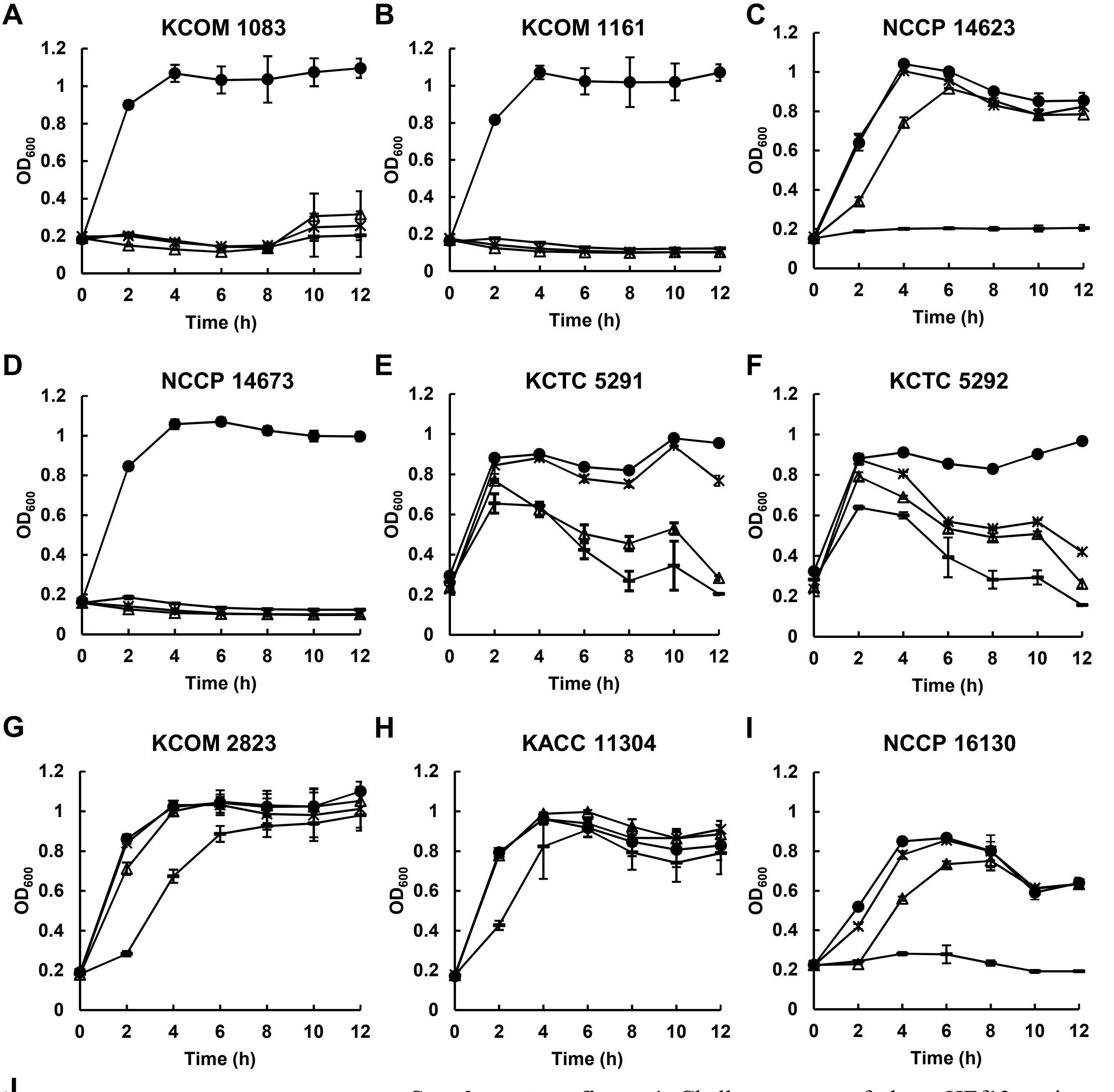

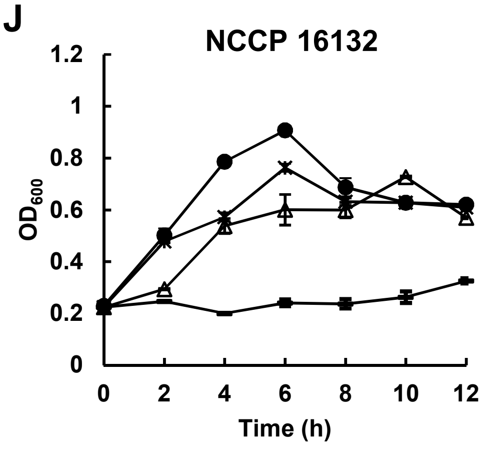

Supplementary figure 1. Challenge assay of phage HEf13 against various *E. faecalis* strains. Bacterial growth of various *E. faecalis* strains showing clear- (A to F), and turbid-plaque formation (G to J) after phage HEf13 infection was measured. Each *E. faecalis* strain was infected with phage HEf13 at various MOIs (0.01 to 1) and incubated for 0-12 h. At the assigned incubation time points, bacterial growth was assessed by measuring optical density at 600 nm (OD_{600}) using a spectrophotometer. Non phage-infected *E. faecalis* strains were used as the negative controls. Values are means ± standard deviations from triplicates of each treatment. Circle, non-treatment; Asterisk, MOI 0.01; Triangle, MOI 0.1; Bar, MOI 1.
