# Supplementary material for: The Novel Enterococcus Phage vB_EfaS_HEf13 Has Broad Lytic Activity Against Clinical Isolates of Enterococcus faecalis
Source: Front Microbiol. 2019 Dec 17;10:2877. doi: 10.3389/fmicb.2019.02877 (PMC6927925; doi:10.3389/fmicb.2019.02877)
Supplement: Supplementary file 2 [file Table_1.DOCX]

**Supplementary table 1.** Summary of comparative genome analysis between phage HEf13 and other *E. faecalis* phages of *Sap6virus* lineage.

| **Phage** | **GenBank accession no.** | **Genome length (kb)** | **GC content (%)** | **Overall DNA sequence identity (%)** | **Query cover** | **Max score** | **No. of CDS** | **No. of tRNAs** |
| --- | --- | --- | --- | --- | --- | --- | --- | --- |
| vB_EfaS_HEf13 | MH618488 | 57,811 | 40.03 | - | - | - | 95 | 1 |
| vB_EfaS_IME198 | KT932699.1 | 58,000 | 40.02 | 95% | 85% | 30406 | 95 | 1 |
| EF-P29 | KY303907.1 | 58,984 | 39.97 | 95% | 84% | 30095 | 101 | 0 |
| VD13 | KJ094032.2 | 55,073 | 40 | 94% | 80% | 28949 | 88 | 2 |
| EF-P10 | KY472224.1 | 49,455 | 39.82 | 95% | 81% | 23769 | 127 | 1 |
| SP-QS1 | HE962497.1 | 58,305 | 39.87 | 96% | 89% | 23769 | 104 | 1 |
| IME-EF1 | KF192053.1 | 57,081 | 40.04 | 95% | 89% | 23704 | 98 | 1 |
| BC-611 | AB712291.1 | 53,996 | 40.45 | 95% | 80% | 18848 | 87 | 1 |
| SAP6 | JF731128.1 | 58,305 | 40 | 95% | 84% | 13514 | 44 | 1 |
