# Supplementary material for: The Novel Enterococcus Phage vB_EfaS_HEf13 Has Broad Lytic Activity Against Clinical Isolates of Enterococcus faecalis
Source: Front Microbiol. 2019 Dec 17;10:2877. doi: 10.3389/fmicb.2019.02877 (PMC6927925; doi:10.3389/fmicb.2019.02877)
Supplement: Supplementary file 3 [file Table_2.DOCX]

**Supplementary table 2.** Annotation of functional ORF in phage HEf13 genome.

| **Locus tag** | **Predicted function** | **Location (nt)** | **BLASTP best matches** | **Identity** | **Percent**  **Identity** |
| --- | --- | --- | --- | --- | --- |
| vBEfaSHEf13_001 | RNA ligase | 148-  1110 | *Streptococcus* phage SPQ-S1 | 247/318 | 78% |
| vBEfaSHEf13_002 | Hypothetical protein | 1112-  1378 | *Enterococcus phage* BC-611 | 85/88 | 97% |
| vBEfaSHEf13_003 | Hypothetical protein | 1371-1619 | *Enterococcus* phage IME-EF1 | 82/82 | 100% |
| vBEfaSHEf13_004 | Hypothetical protein | 1633-1842 | *Enterococcus phage* BC-611 | 69/69 | 100% |
| vBEfaSHEf13_005 | Hypothetical protein | 1832-2050 | *Enterococcus* phage IME-EF1 | 66/72 | 92% |
| vBEfaSHEf13_006 | Hypothetical protein | 2051-2275 | No significant similarity found | | |
| vBEfaSHEf13_007 | Hypothetical protein | 2275-2535 | *Enterococcus phage* BC-611 | 86/86 | 100% |
| vBEfaSHEf13_008 | Hypothetical protein | 2532-2726 | *Enterococcus phage* BC-611 | 62/64 | 100% |
| vBEfaSHEf13_009 | Hypothetical protein | 2805-3236 | *Enterococcus* phage EF-P10 | 116/143 | 74% |
| vBEfaSHEf13_010 | Hypothetical protein | 3249-3449 | *Enterococcus* phage vB_EfaS_IME198 | 65/66 | 100% |
| vBEfaSHEf13_011 | Cytidine deaminase | 3460-4044 | *Enterococcus* phage VD13 | 176/174 | 91% |
| vBEfaSHEf13_012 | Hypothetical protein | 4045-4503 | *Enterococcus* phage IME-EF1 | 103/137 | 75% |
| vBEfaSHEf13_013 | ATP-dependent metalloprotease | 4562-5257 | *Enterococcus* phage IME-EF1 | 221/231 | 96% |
| vBEfaSHEf13_014 | Hypothetical protein | 5250-5642 | *Enterococcus* phage SAP6 | 107/130 | 82% |
| vBEfaSHEf13_015 | Hypothetical protein | 5644-6078 | *Enterococcus* phage vB_EfaS_IME198 | 130/144 | 90% |
| vBEfaSHEf13_016 | DNA polymerase I | 6152-8503 | *Streptococcus* phage SPQ-S1 | 778/783 | 99% |
| vBEfaSHEf13_017 | Hypothetical protein | 8582-8767 | *Enterococcus phage* BC-611 | 58/60 | 97% |
| vBEfaSHEf13_018 | Hypothetical protein | 8767-9144 | *Enterococcus phage* BC-611 | 123/125 | 98% |
| vBEfaSHEf13_019 | Hypothetical protein | 9145-9354 | *Enterococcus* phage EF-P29 | 54/70 | 77% |
| vBEfaSHEf13_020 | Hypothetical protein | 9357-9566 | *Streptococcus* phage SPQ-S1 | 67/69 | 98% |
| vBEfaSHEf13_021 | Hypothetical protein | 9563-9787 | *Enterococcus phage* BC-611 | 70/74 | 95% |
| vBEfaSHEf13_022 | Hypothetical protein | 9802-9996 | *Enterococcus* phage EF-P29 | 57/64 | 89% |
| vBEfaSHEf13_023 | Hypothetical protein | 9997-10176 | *Enterococcus* phage IME-EF1 | 56/59 | 95% |
| vBEfaSHEf13_024 | Hypothetical protein | 10376-10582 | *Enterococcus phage* BC-611 | 62/68 | 91% |
| vBEfaSHEf13_025 | Hypothetical protein | 10594-10710 | *Enterococcus* phage EF-P10 | 38/38 | 100% |
| vBEfaSHEf13_026 | LPS glycosyltransferase | 10713-11276 | *Enterococcus* phage IME-EF1 | 186/187 | 99% |
| vBEfaSHEf13_027 | Hypothetical protein | 11368-12000 | *Enterococcus phage* BC-611 | 209/210 | 99% |
| vBEfaSHEf13_028 | Deoxynucleoside monophosphate kinase | 11993-12562 | *Streptococcus* phage SPQ-S1 | 189/189 | 100% |
| vBEfaSHEf13_029 | Crossover junction endodeoxyribonuclease RuvC | 12559-12993 | *Enterococcus* phage IME-EF1 | 144/144 | 100% |
| vBEfaSHEf13_030 | Hypothetical protein | 13130-13459 | *Enterococcus* phage EF-P29 | 107/109 | 98% |
| vBEfaSHEf13_031 | Hypothetical protein | 13459-14487 | *Enterococcus* phage vB_EfaS_IME198 | 341/342 | 99% |
| vBEfaSHEf13_032 | HNH homing endonuclease | 14480-14920 | *Streptococcus* phage SPQ-S1 | 144/146 | 99% |
| vBEfaSHEf13_033 | Hypothetical protein | 14993-15253 | *Streptococcus* phage SPQ-S1 | 80/86 | 93% |
| vBEfaSHEf13_034 | Hypothetical protein | 15277-15489 | *Enterococcus* phage VD13 | 69/70 | 99% |
| vBEfaSHEf13_035 | DNA methyltransferase | 15455-16207 | *Enterococcus* phage EF-P29 | 250/250 | 99% |
| vBEfaSHEf13_036 | DNA helicase | 16220-17584 | *Streptococcus* phage SPQ-S1 | 454/454 | 100% |
| vBEfaSHEf13_037 | DNA replication protein | 17596-18372 | *Enterococcus phage* BC-611 | 258/258 | 100% |
| vBEfaSHEf13_038 | Transcriptional regulator | 18421-18774 | *Enterococcus* phage IME-EF1 | 117/117 | 100% |
| vBEfaSHEf13_039 | DNA primase | 18849-19793 | *Enterococcus* phage SAP6 | 314/314 | 100% |
| vBEfaSHEf13_040 | Hypothetical protein | 19823-20035 | *Streptococcus* phage SPQ-S1 | 70/70 | 100% |
| vBEfaSHEf13_041 | Hypothetical protein | 20047-20235 | *Streptococcus* phage SPQ-S1 | 62/62 | 100% |
| vBEfaSHEf13_042 | Hypothetical protein | 20401-20733 | *Streptococcus* phage SPQ-S1 | 101/110 | 92% |
| vBEfaSHEf13_043 | Hypothetical protein | 20733-20984 | *Enterococcus* phage EF-P10 | 60/82 | 84% |
| vBEfaSHEf13_044 | Hypothetical protein | 20984-21229 | *Enterococcus* phage VD13 | 50/78 | 73% |
| vBEfaSHEf13_045 | Hypothetical protein | 22038-22250 | *Streptococcus* phage SPQ-S1 | 45/46 | 98% |
| vBEfaSHEf13_046 | Hypothetical protein | 22348-22548 | *Enterococcus* phage IME-EF1 | 63/66 | 95% |
| vBEfaSHEf13_047 | Hypothetical protein | 22548-22934 | *Enterococcus phage* BC-611 | 126/128 | 98% |
| vBEfaSHEf13_048 | Hypothetical protein | 22937-23371 | *Enterococcus* phage IME-EF1 | 144/144 | 100% |
| vBEfaSHEf13_049 | Hypothetical protein | 23424-24248 | *Enterococcus* phage SAP6 | 272/274 | 99% |
| vBEfaSHEf13_050 | HNH homing endonuclease | 24758-24973 | *Enterococcus* phage EF-P29 | 33/57 | 58% |
| vBEfaSHEf13_051 | Hypothetical protein | 24961-25389 | *Streptococcus* phage SPQ-S1 | 141/142 | 100% |
| vBEfaSHEf13_052 | N-acetylmuramoyl-L-alanine amidase | 25673-26386 | *Enterococcus* phage IME-EF1 | 236/237 | 99% |
| vBEfaSHEf13_053 | Hypothetical protein | 26433-26720 | *Enterococcus* phage VD13 | 94/95 | 99% |
| vBEfaSHEf13_054 | Depolymerase | 26734-29760 | *Streptococcus* phage SPQ-S1 | 995/1008 | 99% |
| vBEfaSHEf13_055 | Tail fibers | 29773-33765 | *Enterococcus* phage IME-EF1 | 1324/1330 | 99% |
| vBEfaSHEf13_056 | Tail tape measure protein | 33779-36664 | *Streptococcus* phage SPQ-S1 | 946/961 | 99% |
| vBEfaSHEf13_057 | Hypothetical protein | 36677-36901 | *Streptococcus* phage SPQ-S1 | 74/74 | 100% |
| vBEfaSHEf13_058 | Hypothetical protein | 36912-37352 | *Enterococcus phage* BC-611 | 145/146 | 100% |
| vBEfaSHEf13_059 | Phage tail tube protein | 37496-38185 | *Enterococcus phage* BC-611 | 229/229 | 100% |
| vBEfaSHEf13_060 | Tail protein | 38206-38640 | *Streptococcus* phage SPQ-S1 | 144/144 | 100% |
| vBEfaSHEf13_061 | Hypothetical protein | 38206-38640 | *Streptococcus* phage SPQ-S1 | 126/126 | 100% |
| vBEfaSHEf13_062 | Hypothetical protein | 38653-39033 | *Enterococcus phage* BC-611 | 125/125 | 100% |
| vBEfaSHEf13_063 | Head-tail connector family protein | 39411-39815 | *Enterococcus phage* BC-611 | 132/134 | 99% |
| vBEfaSHEf13_064 | Major tail protein | 39875-40315 | *Enterococcus phage* BC-611 | 145/145 | 100% |
| vBEfaSHEf13_065 | Major capsid protein | 40470-41276 | *Enterococcus phage* BC-611 | 265/268 | 99% |
| vBEfaSHEf13_066 | Hypothetical protein | 41325-41999 | *Enterococcus phage* BC-611 | 224/224 | 100% |
| vBEfaSHEf13_067 | Head morphogenesis protein | 42110-42865 | *Enterococcus* phage EF-P29 | 250/251 | 99% |
| vBEfaSHEf13_068 | Phage portal protein | 42877-44412 | *Enterococcus* phage SAP6 | 510/511 | 99% |
| vBEfaSHEf13_069 | Phage terminase large subunit | 44469-45740 | *Streptococcus* phage SPQ-S1 | 423/423 | 100% |
| vBEfaSHEf13_070 | Holin | 45803-46051 | *Enterococcus phage* BC-611 | 82/82 | 100% |
| vBEfaSHEf13_071 | Hypothetical protein | 46070-46414 | *Enterococcus phage* BC-611 | 114/114 | 100% |
| vBEfaSHEf13_072 | Phage terminase small subunit | 46428-47027 | *Enterococcus phage* BC-611 | 199/199 | 100% |
| vBEfaSHEf13_073 | Hypothetical protein | 47308-47622 | *Enterococcus* phage vB_EfaS_IME198 | 104/104 | 100% |
| vBEfaSHEf13_074 | Hypothetical protein | 47622-47879 | *Enterococcus* phage IMEEF1 | 84/85 | 95% |
| vBEfaSHEf13_075 | DNA Methyltransferase | 47872-48324 | *Enterococcus* phage IMEEF1 | 116/126 | 92% |
| vBEfaSHEf13_076 | Hypothetical protein | 48328-48465 | *Streptococcus* phage SPQ-S1 | 45/45 | 100% |
| vBEfaSHEf13_077 | Glutaredoxin-like protein | 48490-48750 | *Enterococcus* phage vB_EfaS_IME198 | 84/86 | 100% |
| vBEfaSHEf13_078 | Hypothetical protein | 48752-48958 | *Enterococcus* phage IME-EF1 | 60/68 | 88% |
| vBEfaSHEf13_079 | Hypothetical protein | 48948-49250 | *Enterococcus* phage IME-EF1 | 63/100 | 63% |
| vBEfaSHEf13_080 | Hypothetical protein | 49270-49641 | *Enterococcus* phage EF-P29 | 106/123 | 86% |
| vBEfaSHEf13_081 | Hypothetical protein | 50092-50757 | *Enterococcus* phage IME-EF1 | 211/221 | 95% |
| vBEfaSHEf13_082 | Hypothetical protein | 50742-51626 | *Enterococcus* phage SAP6 | 248/284 | 87% |
| vBEfaSHEf13_083 | Hypothetical protein | 51627-51845 | *Enterococcus phage* BC-611 | 70/72 | 98% |
| vBEfaSHEf13_084 | Hypothetical protein | 51847-52059 | *Enterococcus* phage EF-P10 | 68/70 | 97% |
| vBEfaSHEf13_085 | Hypothetical protein | 52236-52376 | *Enterococcus phage* BC-611 | 45/46 | 98% |
| vBEfaSHEf13_086 | Hypothetical protein | 53500-53802 | *Enterococcus* phage vB_EfaS_IME198 | 92/100 | 96% |
| vBEfaSHEf13_087 | Hypothetical protein | 54054-54488 | *Enterococcus* phage IME-EF1 | 137/144 | 95% |
| vBEfaSHEf13_088 | Hypothetical protein | 54625-54879 | *Enterococcus phage* BC-611 | 83/84 | 99% |
| vBEfaSHEf13_089 | Hypothetical protein | 54948-55082 | *Streptococcus* phage SPQ-S1 | 39/44 | 89% |
| vBEfaSHEf13_090 | Hypothetical protein | 55161-55535 | *Enterococcus* phage SAP6 | 122/124 | 98% |
| vBEfaSHEf13_091 | Hypothetical protein | 55528-55914 | *Enterococcus* phage EF-P29 | 127/128 | 99% |
| vBEfaSHEf13_092 | Hypothetical protein | 56015-56254 | No significant similarity found | | |
| vBEfaSHEf13_093 | Hypothetical protein | 56540-56653 | No significant similarity found | | |
| vBEfaSHEf13_094 | Hypothetical protein | 57112-57624 | No significant similarity found | | |
| vBEfaSHEf13_095 | DNA binding protein | 57627-57809 | *Enterococcus* phage EF-P29 | 57/60 | 95% |
